# Cell-Autonomous Defects in Thymic Epithelial Cells Disrupt Endothelial-Perivascular Cell Interactions in the Mouse Thymus

**DOI:** 10.1371/journal.pone.0065196

**Published:** 2013-06-04

**Authors:** Jerrod L. Bryson, Ann V. Griffith, Bernard Hughes III, Fumi Saito, Yousuke Takahama, Ellen R. Richie, Nancy R. Manley

**Affiliations:** 1 Department of Cellular Biology, University of Georgia, Athens, Georgia, United States of America; 2 Department of Carcinogenesis, University of Texas, M.D. Anderson Cancer Center, Science Park Research Division, Smithville, Texas, United States of America; 3 Department of Genetics, University of Georgia, Athens, Georgia, United States of America; 4 Division of Experimental Immunology, Institute for Genome Research, University of Tokushima, Tokushima, Japan; Brigham and Women’s Hospital, United States of America

## Abstract

The thymus is composed of multiple stromal elements comprising specialized stromal microenvironments responsible for the development of self-tolerant and self-restricted T cells. Here, we investigated the ontogeny and maturation of the thymic vasculature. We show that endothelial cells initially enter the thymus at E13.5, with PDGFR-β^+^ mesenchymal cells following at E14.5. Using an allelic series of the thymic epithelial cell (TEC) specific transcription factor Foxn1, we showed that these events are delayed by 1–2 days in *Foxn1* ^Δ*/*Δ^ mice, and this phenotype was exacerbated with reduced Foxn1 dosage. At subsequent stages there were fewer capillaries, leaky blood vessels, disrupted endothelium - perivascular cell interactions, endothelial cell vacuolization, and an overall failure of vascular organization. The expression of both *VEGF-A* and *PDGF-B,* which are both primarily expressed in vasculature-associated mesenchyme or endothelium in the thymus, were reduced at E13.5 and E15.5 in *Foxn1* ^Δ*/*Δ^ mice compared with controls. These data suggest that Foxn1 is required in TECs both to recruit endothelial cells and for endothelial cells to communicate with thymic mesenchyme, and for the differentiation of vascular-associated mesenchymal cells. These data show that Foxn1 function in TECs is required for normal thymus size and to generate the cellular and molecular environment needed for normal thymic vascularization. These data further demonstrate a novel TEC-mesenchyme-endothelial interaction required for proper fetal thymus organogenesis.

## Introduction

Organ vascularization is essential for the delivery of oxygen and nutrients to developing tissues and is required for normal tissue growth and homeostasis. Vasculature is primarily comprised of endothelial and perivascular support cells [1]. In addition to their essential role in providing oxygen and nutrients to tissues, recent reports have highlighted the versatility of the developing endothelia in organs [2]. Several groups have demonstrated that the vascular endothelium serves unique organ-specific functions in the early organogenesis of the liver, lung, pancreatic islets, and kidney [3], , and initial formation of vascular networks has been described in a number of organ systems.

In the thymus, the vascular network plays a critical role in organ function, and has also been implicated in organ development. The thymus provides a specialized microenvironment that supports the development of self-MHC restricted and self-tolerant T cells. Lymphoid progenitor cells (LPCs) enter the thymus and CD4^+^ and CD8^+^ single positive T cells exit to the periphery in a highly regulated fashion via specialized blood vessels at spatially defined regions of the organ [6], [7]. The postnatal thymus is comprised predominantly of hematopoietic-derived cells (mainly thymocytes, but also dendritic cells and macrophages), in close association with a complex network of non-hematopoietic-derived stromal cells (epithelial, mesenchymal, and endothelial cells) [8].

The predominant functional stromal cell population is the thymic epithelial cell (TEC) compartment. TECs are broadly subdivided into cortical (cTEC) and medullary (mTEC) subsets, which have specific roles promoting the development of T cells [9]. The organization of the thymic stroma into cortical and medullary compartments is critical for correct and efficient production of developing T cells, and the thymic vasculature has been implicated in regulating this organization [10]. In spite of these essential functional roles, cellular and molecular mechanisms regulating the initial establishment of a functional thymic vasculature are poorly understood.

As in all organs, initial thymic vascularization takes place during fetal organ development. The fetal thymus and parathyroid glands develop from an outgrowth of the third pharyngeal pouch endoderm [11], [12]. Between E10.5–11.5, neural crest cell (NCC)-derived mesenchymal cells condense around the third pharyngeal pouch and contribute to the progression of thymic organogenesis by providing patterning signals to the developing epithelium [13]. LPCs first immigrate into the thymus at E11.5, by direct migration across the developing capsule in response to chemokines made by the endodermally-derived primordium [14]. NCC-derived mesenchyme contributes to later organogenesis and fetal development by forming the thymic capsule, which provides growth signals to the developing thymic rudiment [15] and perivascular support cells that stabilize the developing vasculature [16], [17]. The thymic vasculature is connected to the peripheral vasculature by E15.5 [14]. In the postnatal thymus, LPCs enter the thymus via postcapillary venules (PCV) positioned at the cortical medullary junction (CMJ), while mature single positive (SP) T cells typically co-opt larger vessels positioned at the CMJ as a route of thymic egress [6], [18]. Correct formation and patterning of the thymic vascular network is therefore critical for postnatal thymic function.

Two recent studies have reported on the role of VEGF in the thymus of neonatal and adult mice [19], [20]. In one study, VEGF-A was deleted in the thymic epithelium using a nude mouse blastocyst complementation strategy [20]. Analysis of these postnatal thymi showed altered thymic vascular network formation, but normal CD4:CD8 ratios in the thymus and periphery [20]. Thymic cortical mesenchymal cells were also identified as a source of VEGF-A, suggesting that the coordinated expression of VEGF-A in TECs and mesenchyme contributes to the formation of thymic blood vessels [20]. In another study, thymic vascular networks were compared in neonatal and adult mice. The neonatal thymus expressed increased levels of VEGF-A relative to adult thymus, and contained dense, highly branched capillary networks lacking significant perivascular cell coverage, characteristic of immature vascular structures [19]. In contrast, adult thymi expressed low levels of VEGF-A, exhibited less vessel branching, and increased expression of perivascular cell markers, suggestive of a mature vascular network [19]. These reports emphasize the importance of VEGF-A expression in the establishment of the postnatal thymic vasculature. However, neither of these studies investigated fetal thymus phenotypes, and the molecular and cellular control of initial thymic vascularization remains to be determined.

Thymic epithelial cells express the forkhead box transcription factor Foxn1 at E11.25, just before colonization of the thymus by the first wave of thymocytes [21], [22]. Foxn1 is required cell-autonomously for both cTEC and mTEC differentiation [23], [24], and plays a central role in multiple aspects of TEC development and organization. In the absence of Foxn1 (nude mice), lymphoid progenitor immigration is not supported [14], [25], and endothelial progenitor and NCC-derived mesenchymal cells fail to enter the thymic rudiment [26]. However, given the general failure of both TEC differentiation and of recruitment of cells into the nude thymic rudiment, it is not possible to determine from analysis of the null allele alone what role TECs play in the formation of the intrathymic vasculature. We previously reported the generation of a hypomorphic allele of *Foxn1*, referred to as *Foxn1* ^Δ^ [27], in which an N-terminal domain of the protein is deleted, while maintaining the DNA-binding and acidic activation domains. This allele results in specific changes in Foxn1-dependent functions, causing phenotypes that are less severe than the null allele. In *Foxn1* ^Δ*/*Δ^ homozygotes, TEC differentiation is largely arrested at an immature stage, and organization of defined cortical and medullary epithelial cell compartments fails. These mice therefore provide a model for investigating the roles of Foxn1 in later stages of fetal thymus organogenesis [27], [28].

To test whether proper TEC differentiation is required for vascular development during thymus organogenesis, we analyzed an allelic series including *Foxn1^+/^* ^Δ^, *Foxn1* ^Δ*/*Δ^, *Foxn1* ^Δ*/nu*^ and *Foxn1^nu/nu^* embryos. Initial attraction of the embryonic vasculature and neural crest cells (NCCs) to the vicinity of the thymus rudiment and initial immigration of lymphoid progenitor cells (LPCs) into the thymus are normal until E12.5 in *Foxn1^+/^* ^Δ^ and *Foxn1* ^Δ*/*Δ^ mice. However, there was a specific delay in endothelial and neural crest-derived perivascular cell recruitment into the thymus in *Foxn1* ^Δ*/*Δ^ mice. Progressive exacerbation of this vascular phenotype in *Foxn1* ^Δ*/nu*^ and *Foxn1^nu/nu^* mice demonstrated that the process of thymus vascularization is sensitive to changes in *Foxn1* function and dosage. We further demonstrated that the thymic blood vessel network is connected to the fetal vascular network at E14.5 in both control and *Foxn1* ^Δ*/*Δ^ mice. However, blood vessel patterning was compromised in *Foxn1* ^Δ*/*Δ^ thymi, as indicated by “leaky” vessels, an apparent decrease in capillaries, loss of tight association between the endothelium and perivascular cells, indistinct vessel walls, vacuolated endothelium, and an overall failure of vessels to model into a stereotypical thymus vascular network. These results demonstrate that Foxn1-expressing thymic epithelial cells are required for normal epithelial-endothelial-mesenchymal cell interactions that are necessary for the formation of a functional vascular network in the thymus.

## Methods

### Mice

*Foxn1* ^Δ^ mice were previously described [27]. *Foxn1* ^Δ^ mice are on a mixed 129v/C57BL/6J background that has been backcrossed to C57BL/6J for 5 to 7 generations. *Foxn1^nu^* mice on a C57BL/6J background were purchased from The Jackson Laboratories (Bar Harbor, ME). *Foxn1* ^Δ*/nu*^ mice were generated from *Foxn1* ^Δ*/*Δ^ X *Foxn1^+/nu^* crosses. All experiments were performed with approval from the UGA Institutional Animal Care and Use Committee (approval number 04-003).

### Immunofluorescence

Embryos were isolated at E11.5–E18.5, flash frozen in liquid nitrogen and cryosectioned at 10 µm. The following primary antibodies were used for the embryonic thymus immunofluorescence analysis [14], [27]: monoclonal rat CD31 and CD144 (BD Pharmingen, 1∶100), goat PDGFR-ß (1∶50), goat VEGF (15 µg/mL), rat CCL21, and rat CCL25 (R&D Systems), rat CD45 (eBioscience, 1∶100), mouse polyclonal Keratin 5 (Covance, 1∶500), mouse Cytokeratin (Sigma, 1∶400), rabbit Collagen IV (1∶400). The following secondary antibodies were purchased from Jackson ImmunoResearch: α-mouse CY5, α-rat FITC, α-goat Texas Red, α-rabbit CY3, and SA-FITC. α-rat Alexa 488 and α-goat 633 were purchased from Invitrogen. For CD144 staining we used the TSA Biotin System (PerkinElmer) for signal amplification, followed by incubation with streptavidin-FITC (Jackson ImmunoResearch, 1∶100). Fluorescence images were collected using either confocal microscope (LSM 510 Meta, Zeiss) and captured using a Plan-Apochromat 20×/0.8 objective or Axioplan (Zeiss) microscope and AxioVision 4.8 software. All images are representative of at least three independent experiments. N values for each experiment are indicated in the text and figure legends.

### Thymic Stromal Cell Isolation

Thymi from E13.5 and E15.5 embryos were dissected and digested in 0.25% trypsin. Mutant and control littermates were pooled separately after genotyping yolk sac DNA. Following digestion, cells were incubated with purified mouse anti-mouse CD45 (BioLegend) for 30 min and washed three times with PBS. CD45^+^ cells were depleted using Dynabeads® sheep anti-mouse and following manufacture’s protocol for magnetic bead depletion (Invitrogen, USA). CD45 purity and EpCAM expression was determined by real-time quantitative polymerase chain reaction (qRT-PCR) (Figure S1).

### RNA Isolation, cDNA Synthesis and Real-time Quantitative Reverse Transcriptase Polymerase Chain Reaction (qRT-PCR)

RNA was isolated from embryonic thymi using the RNeasy Micro kit (QIAGEN). In each litter, mutant thymi were pooled, as well as control thymi. First-strand cDNA was synthesized using a cDNA synthesis kit (Bio-Rad, USA). qRT-PCR was performed using TaqMan Universal Master Mix and TaqMan probes for VEGF-A, PDGF-B, EpCAM, CD45 and 18S rRNA (endogenous control) on an ABI 7500 thermocycler. Controls are set to a value of 1 in each experiment relative to mutants. All experiments were performed in duplicate and analyzed using the ΔC_T_ method.

### *In vivo* Embryonic Thymic Vasculature Labeling

Fetal vascular labeling was performed as previously described [29].

### Ultrastructural Analysis of Thymus Vasculature

Thymi were fixed and processed for electron microscopy as previously described [30]. Images were captured using the JEOL JEM-1210 Transmission Electron Microscope.

### Image Analysis

Average mean fluorescence intensity was calculated using Zeiss LSM 510 software. CD31^+^ Area/Thymus Area was calculated using CellProfiler cell image analysis software [31].

### Statistics

Values are expressed as means plus or minus Standard Deviation (SD). Student’s t test was performed to determine whether the difference between the means of mutants compared to control groups were statistically significant.

## Results

### Timing and Characteristics of Normal Vasculature Ontogenesis

**Figure 1:**
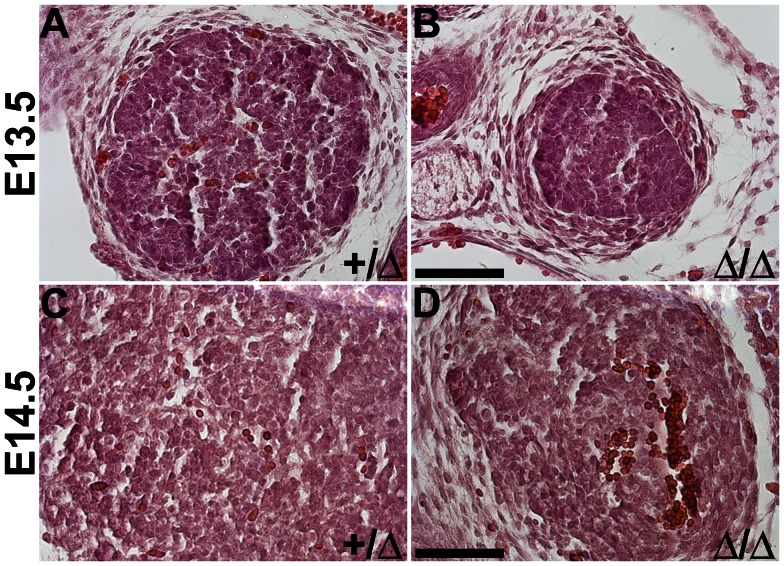
Histological Analysis of vasculature in embryonic thymus of *Foxn1^Δ/Δ^* Mice. Haematoxylin & Eosin staining on paraffin section of fetal thymus (E13.5–E14.5). (**A**) E13.5 *Foxn1^+/Δ^* embryonic thymus with RBC detected throughout rudiment (**B**) Absence of RBC in E13.5 *Foxn1^Δ/Δ^* embryonic thymus (**C**) RBC present in E14.5 *Foxn1^+/Δ^* and (**D**) *Foxn1^Δ/Δ^* embryonic thymus. Scale bar = 50 µm; n = 3.

**Figure 2:**
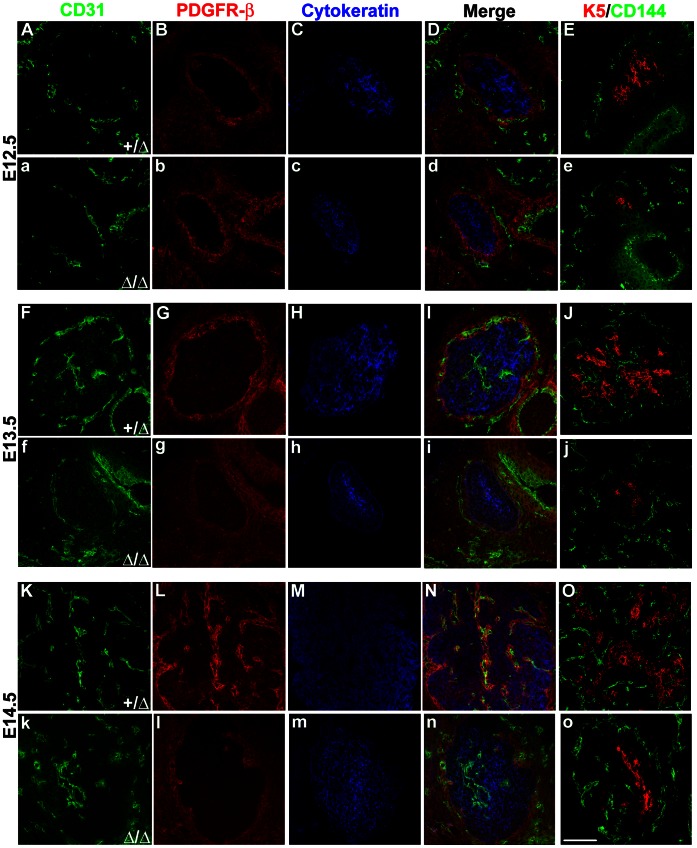
Initial embryonic thymic vascularization is defective in *Foxn1^Δ/Δ^* Mice. Immunostaining on frozen transverse sections of fetal thymus (E12.5–E14.5). Endothelial and stromal cell markers used are listed above each column in the corresponding color: CD31^+^/CD144^+^ for endothelial cells (**green**); PDGFR-β^+^ for neural crest mesenchyme (**red**); Cytokeratin (**blue**) or Keratin 5 (**red, K5**) for epithelial cells. Embryonic stages in the first column and genotypes to the left apply to the entire row unless otherwise labeled. (**A–e**) CD31^+^/CD144^+^ endothelial cells and PDGFR-β^+^ neural crest cells are present in the thymic capsule region in *Foxn1^+/Δ^* (**A–E**) and *Foxn1^Δ/Δ^* mice (**a–e**) at E12.5. (**F–o**) CD31^+^/CD144^+^ cells followed by PDGFR-β^+^ cells initially immigrate into the thymus at E13.5 in heterozygotes (**F–J**) and at E14.5 in homozygotes (**k–o**). Scale bar, 50 µm; n = 3.

**Figure 3:**
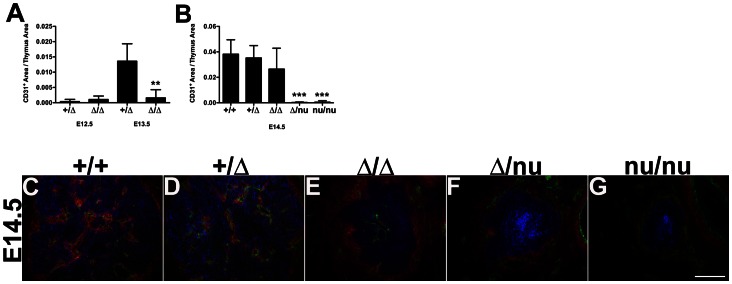
Thymic vascularization is sensitive to Foxn1 levels. Quantification of CD31^+/^Thymus Area from immunostained frozen sections of embryonic thymi (**A**) E12.5 Foxn1^+/Δ^ (n = 8) and Foxn1^Δ/Δ^ (n = 6); p>0.05 and E13.5 Foxn1^+/Δ^ (n = 5) and Foxn1^Δ/Δ^ (n = 7); p<0.001. (**B**) E14.5 Foxn1^+/+^ (n = 7), Foxn1^+/Δ^ (n = 8); p>0.05, Foxn1^Δ/Δ^ (n = 10); p>0.05, Foxn1^Δ/nu^ (n = 9); p = <0.0001, Foxn1^nu/nu^ (n = 6); p<0.0001. (**C–G**) CD31^+^ endothelial cells (**green**) and PDGFR-β^+^ neural crest mesenchyme (**red**) can be detected in the thymic capsule and inside the keratin-positive thymus (**blue**) in E14.5 (**C**) *Foxn1^+/+^* (**D**) *Foxn1^+/Δ^* (**E**) *Foxn1^Δ^* ^/*Δ*^ (**F**) but in the capsule only in *Foxn1^Δ/nu^* (**G**) and *Foxn1^nu/nu^* mice. Scale bar, 100 µm; n = 3.

To identify initial stages of thymus vascularization, we first performed a histological analysis of the embryonic thymus and assayed for the presence of morphologically apparent blood vessels containing red blood cells (RBCs) in the rudiment (Figure 1). Vessels containing RBC were detected as early as E13.5 in the *Foxn1^+/^* ^Δ^ thymus (Figure 1A), but were noticeably absent in *Foxn1* ^Δ*/*Δ^ littermates (Figure 1B). RBCs were detected in both E14.5 *Foxn1^+/^* ^Δ^ and *Foxn1* ^Δ*/*Δ^ thymi (Figure 1C–D). We next assayed E12.5–E14.5 *Foxn1^+/^* ^Δ^ and *Foxn1* ^Δ*/*Δ^ embryonic thymi for the presence of two endothelial cell markers, PECAM-1 (CD31) and VE-Cadherin (CD144). We also confirmed that the timing of vascularization events was similar between wild-type and *Foxn1^+/^* ^Δ^ embryos (Figure S2), confirming that the heterozygotes were appropriate controls for these events. CD31^+^ and CD144^+^ endothelial cells surrounded the thymic epithelial rudiment by E12.5 (Figure 2A, D–E, and 3A), just outside of the PDGFRβ^+^ neural crest-derived mesenchymal capsule that encompasses the thymic rudiment [13], [16], [17], [18]. By E13.5, a network of nascent vascular endothelial structures were present within the thymus and associated with the centrally localized Keratin 5^+^ (K5) subset of thymic epithelial cells (Figure 2F, I–J and 3A). Endothelial cells also remained associated with PDGFR-β^+^ cells in the perithymic mesenchyme (Figure 2F, G, and I) [14]. At E14.5, the vascular network was markedly more complex, with an increased number of CD31^+^ and CD144^+^ cells within the thymus (Figure 2K, N–O and 3B–D). At this stage PDGFR-β^+^ cells had migrated into the thymus in close association with the vascular network (Figure 2N). PGDFR-β^+^ cells in the capsule also began to migrate into the TEC network at E14.5 (Figure 2L, N and 3C–D). These data are consistent with a model in which PDGFR-β^+^ mesenchyme and endothelial cells first encapsulate the E12.5 thymus, and that vascular branches subsequently penetrate the thymus capsule, followed by PDGFR-β^+^ mesenchymal cells migrating along the endothelium into the thymic rudiment.

### Vascularization and NCC Immigration are Delayed in the Foxn1^Δ/Δ^ Thymus

To test whether Foxn1-dependent TEC differentiation is required for initial vascular development; we analyzed mice homozygous for the hypomorphic *Foxn1* ^Δ^ allele. At E12.5, the *Foxn1* ^Δ*/*Δ^ thymic rudiment resembled the stage-matched *Foxn1^+/^* ^Δ^ control littermate thymus (Figure 2A–E, a-e, and 3A). However, at E13.5 CD31^+^ and CD144^+^ endothelium remained at the periphery of the *Foxn1* ^Δ*/*Δ^ thymic epithelial primordium (Figure 2f, h–j and 3A). By E14.5, CD31^+^ and CD144^+^ endothelium in Foxn1^Δ/Δ^ mice was observed in the thymus (Figure 2k, m-o, 3B and E), at similar frequencies compared to control littermates (Figure 2K, M–O, and 3B–D). Moreover, we observed only occasional PDGFR-β^+^ cells in E14.5 *Foxn1* ^Δ*/*Δ^ thymi (Figure 2l, n, and 3E) compared to E14.5 controls (2L, N, and 3C–D), and most endothelial cells in the mutant thymus were not associated with mesenchyme at this stage (Figures 2l, n; 3E). Overall, the localization of endothelial and neural crest cells in E14.5 *Foxn1* ^Δ*/*Δ^ mutant thymi appeared similar to E13.5 controls, indicating a one-day delay in initial thymic vascularization.

### Initial Thymic Vascularization is Sensitive to Foxn1 Function in TECs

We recently demonstrated that the postnatal thymic microenvironment is exquisitely dependent on *Foxn1* dose to maintain postnatal thymic architecture and function [32]. To determine whether the initial establishment of thymic vasculature was sensitive to *Foxn1* function, we combined our Δ allele and the null allele, nude (*nu*), and assayed for vascular markers in *Foxn1* ^Δ*/*Δ^, *Foxn1* ^Δ*/nu*^, and *Foxn1^nu/nu^* mice (Figure 3B–G). The delay in vascular immigration seen in the *Foxn1* ^Δ*/*Δ^ thymus was more pronounced in *Foxn1* ^Δ*/nu*^ (Figure 3F) and *Foxn1^nu/nu^* mice (Figure 3G), with both endothelium and NCCs restricted to the capsule at E14.5 (Figure 3B, E–G). In the absence of Foxn1 (*nu/nu*), CD144^+^ endothelium was not present inside the thymic epithelial rudiment at least until E17.5 (data not shown). We also noted that although initial capsule formation as assayed by PDGFR-β expression appeared normal (Figure 2D, d) the density of the NCC-capsule and/or its expression of PDGFR-β decreased in *Foxn1* ^Δ*/*Δ^ mutants from E13.5 (Figure 2I, i, N, n) and was further reduced in *Foxn1* ^Δ*/nu*^ and *Foxn1^nu/nu^* mice (Figure 3E–G). These data confirm that Foxn1 is required in a dose-dependent manner for TEC number and differentiation. These data further suggest that these TEC-intrinsic changes in thymus size and/or maturation affect vascularization of the thymus primordium during organogenesis by directly or indirectly affecting the NC cells and endothelial cells.

### Timing of Initial LPC Immigration into Foxn1^Δ/Δ^ Thymi is Normal

**Figure 4:**
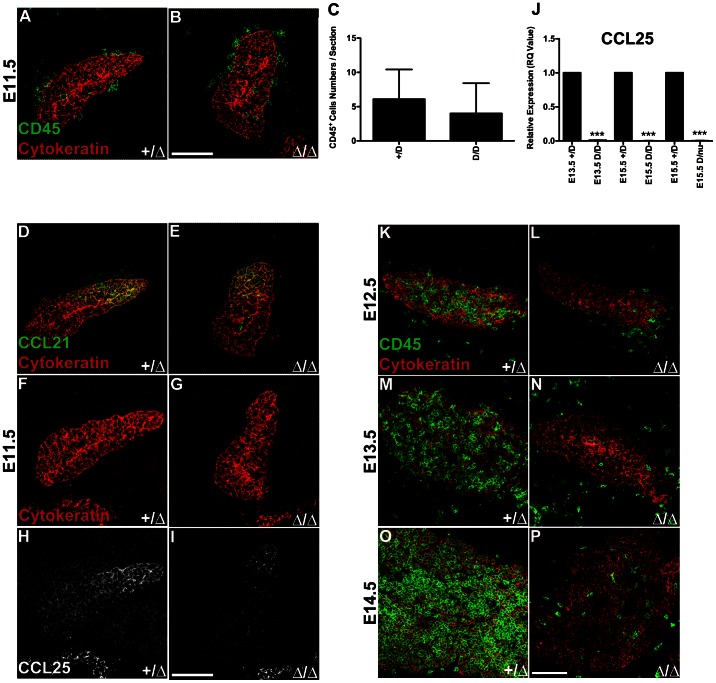
Initial LPC thymic immigration is normal in *Foxn1^Δ^* mice. (**A**) CD45^+^ LPCs (**green**) colonize the *Foxn1^+/Δ^* and (**B**) *Foxn1^Δ/Δ^* thymus at E11.5. (**C**) At E11.5, the frequency of CD45^+^ cells/section was similar between *Foxn1^+/Δ^* (n = 10) and *Foxn1^Δ/Δ^* (n = 9) thymi; (p>0.05). (**D**) Immunostaining for CCL21 (**green**) expression is similar in *Foxn1^+/Δ^* and (**E**) *Foxn1^Δ/Δ^* mouse thymus. (**F–I**) Reduced expression of CCL25 (**white**) in (**G** and **I**) *Foxn1^Δ/Δ^* compared to (**F** and **H**) *Foxn1^+/Δ^* thymus at E11.5. Cytokeratin (**red**). (**J**) CCL25 expression was significantly reduced in E13.5 *Foxn1^Δ^* ^/*Δ*^ **(**n = 4), E15.5 *Foxn1^Δ^* ^/*Δ*^ (n = 3), and E15.5 *Foxn1^Δ^* ^/nu^ (n = 6), compared to *Foxn1^+^* ^/*Δ*^ control thymi. CD45^+^ cells (**green**) were noticeably reduced in (**K–L**) E12.5, (**M–N**) E13.5, and (**O–P**) E14.5 *Foxn1^Δ/Δ^* thymi compared to control littermates. Scale bar, 100 µm. qRT experiments represent relative RNA expression of pooled thymi. Controls were set to 1. Asterisks denote statistical significance.

We previously reported that *Foxn1* ^Δ*/*Δ^ mice have a significant decrease in total thymocytes at both fetal and adult stages [27]. In nude mice (*Foxn1^nu/nu^*), bone marrow derived-hematopoietic precursor cells (HPC) migrate to, but fail to colonize the thymus rudiment [14]. We therefore tested whether the *Foxn1* ^Δ^ mutation affected the timing of initial HPC infiltration of the thymus, which might contribute to these early vascular defects in *Foxn1* ^Δ*/*Δ^ mice. At E11.5, when thymocytes initially infiltrate the thymus [22], CD45^+^ cells were present in the thymus rudiment in both *Foxn1* ^Δ*/*Δ^ and control littermates at similar frequencies (Figure 4A–B and C). At E12.5–E14.5, CD45^+^ cells were significantly reduced in the mutant thymus, consistent with our previous results [27](Figure 4K–P). These data suggest that although CD45^+^ cells in the thymus are reduced after E11.5, the timing of initial HPC colonization of the thymus is normal in *Foxn1* ^Δ*/*Δ^ mice.

We further tested whether CCL21 and CCL25, two key chemokines required for initial HPC recruitment to the shared thymus/parathyroid primordia, were reduced in *Foxn1* ^Δ*/*Δ^ mice at E11.5 [14]. CCL25 is predominantly *Foxn1*-dependent, whereas CCL21 is parathyroid-associated and *Gcm2*-dependent [14]. As expected, CCL21 expression in the Gcm2 domain was normal in E11.5 *Foxn1* ^Δ*/*Δ^ thymi (Figure 4E and D). In contrast, CCL25 expression in the thymus domain was dramatically reduced at E11.5 (Figure 4G, I and F, H), E13.5 and E15.5 in *Foxn1* ^Δ*/*Δ^ and *Foxn1* ^Δ*/nu*^ thymi (4J). As mutation of the CCL25 receptor, CCR9, exhibits a ∼40% reduction in the number of initially immigrating thymocytes, but does not affect timing of initial immigration [14], this result was consistent with our conclusion that the timing of initial LPC immigration was normal in *Foxn1* ^Δ*/*Δ^ mice. After this initial immigration, subsequent proliferation and differentiation of thymocytes is strongly suppressed, presumably by delayed and defective TEC differentiation, as we previously published [33], [34], [35].

### Peripheral Circulation Connects to the Thymus at E14.5

**Figure 5:**
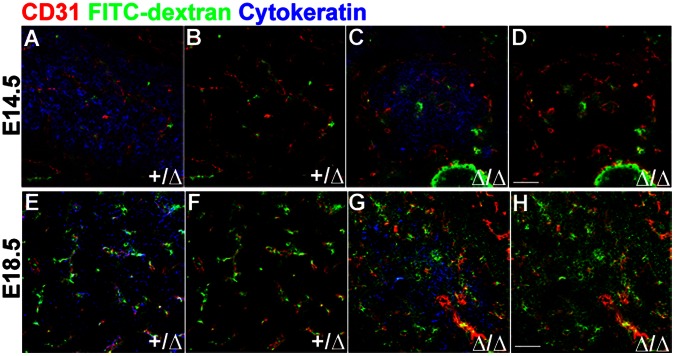
Peripheral circulation is connected to the thymus at E14.5. FITC-dextran (**green**) facial vein injections and immunostaining for CD31 (**red**) and cytokeratin (**blue**) on frozen sagittal sections of fetal mouse thymus. (**A–B**) FITC-dextran is detected in E14.5 *Foxn1^+/Δ^* thymi, tightly associated with CD31^+^ blood vessels. (**C–D**) In *Foxn1^Δ/Δ^* embryos, FITC-dextran is also present, but the signal is more diffusely associated with vessels. (**E–F**) FITC-dextran is present throughout E18.5 *Foxn1^+/Δ^* thymi tightly associated with branched blood vessels. (**G–H**) FITC-dextran is more diffusely present in the thymus of E18.5 *Foxn1^Δ^* ^/*Δ*^ mice. Scale bar, 100 µm; n = 3.

A critical event during thymic organogenesis is the connection of the peripheral vasculature and circulation to the developing vessels in the thymic anlage. This developmental time point likely indicates a switch in the route of LPC entry into the thymus, from initial trans-capsular migration to vascular extravasation at late fetal and postnatal stages [6], [22], [25], [36]. As endothelial cells initially immigrate into the fetal thymus at E13.5, the external connection must occur after this point. To identify the developmental time point at which the peripheral circulation connects to the thymus, we performed FITC-dextran facial vein injections at E14.5. FITC-dextran was detected in *Foxn1^+/^* ^Δ^ thymi tightly associated with CD31^+^ vasculature (Figure 5A–B). The timing of intrathymic FITC-dextran detection was similar in *Foxn1* ^Δ*/*Δ^ mutants (Figure 5C–D), although fewer vessels were labeled in the mutants, and the association of dye with vascular cells appeared looser. This result was consistent with previous data demonstrating the connection is established by E15.5 [14], and suggested that timing of this connection to the embryonic vasculature was regulated by Foxn1-independent mechanisms.

While the timing of vessel connection was similar, vessel patterning and integrity was considerably different. Control thymi showed a network of mostly capillaries throughout the thymus at this stage (Figure 5A–B), while mutant thymi had large, centrally localized vessels with few capillaries between them and the periphery of the rudiment (Figure 5C–D). In all cases, the peripherally injected FITC-dextran remained tightly associated with CD31^+^ and PDGFRβ^+^ vasculature. We observed similar results at E18.5 (Figure 5E–H), in which a dense network of branched blood vessels was present throughout control thymi (Figure 5E–F), with a less dense and branched blood vessel network in the E18.5 *Foxn1* ^Δ*/*Δ^ thymus (Figure 5G–H). Furthermore, at both stages mutants displayed a ‘leaky vessel’ phenotype, in which FITC-dextran within the thymus was not retained within the CD31^+^ and PDGFRβ^+^ vasculature, instead spreading into the surrounding tissue; this phenotype was present at E14.5, but was especially obvious at E18.5 (Figure 5C–D, G–H). These experiments reveal that the peripheral circulation connects to the thymus by E14.5 within 24 hours of initial detection of vasculature-associated markers within the thymus. Furthermore, the timing of this process was normal in *Foxn1* ^Δ*/*Δ^ mutants, but the patterning and maturation of developing vessels was dramatically altered.

### Vascular Patterning is Defective in Late Embryonic and Postnatal Foxn1^Δ/Δ^ Thymus

**Figure 6:**
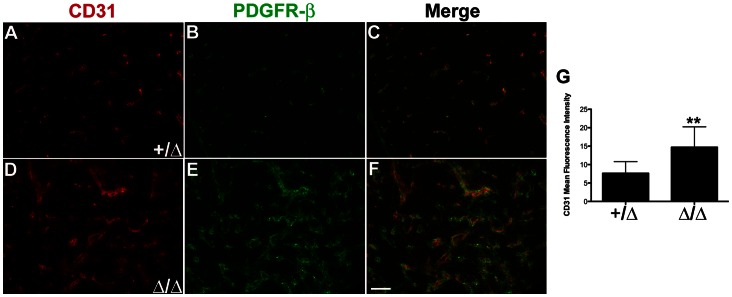
Thymus vascular patterning altered in Foxn1Δ mice. Immunofluorescence analysis on frozen sagittal sections of *Foxn1* ***^+^*** ^/*Δ*^ and *Foxn1^Δ^* ^/*Δ*^ newborn thymus for CD31^+^ (**red**) and PDGFR-β^+^ (**green**) cells in (**A–C**) *Foxn1* ***^+^*** ^/*Δ*^ and (**D–F**) *Foxn1^Δ^* ^/*Δ*^ mice. (**G**) Average mean fluorescence intensity for CD31 in *Foxn1^+^* ^/*Δ*^ (n = 8) *Foxn1^Δ^* ^/*Δ*^ (n = 6) thymus sections. Asterisks denote statistical significance (P<0.05). Scale bar, 50 µm; n = 3.

Thymic vascular patterning requires the formation of a primary blood vessel network and subsequent vascular pruning. This process culminates with the establishment of a capillary-rich cortex that connects to PCVs at the CMJ and within the medulla (Figure 6A–C). However, patterning of the thymus vascular network was defective in newborn *Foxn1* ^Δ*/*Δ^ mutants (Figure 6D–F), which displayed medium-large sized blood vessels throughout the organ, compared to controls. This apparent increase in CD31^+^ vasculature in mutant thymi was statistically significant (p<0.05), as shown by comparing the average mean fluorescence intensity measurements (Figure 6G) from *Foxn1^+/^* ^Δ^ (n = 8) and *Foxn1* ^Δ*/*Δ^ (n = 6) mutants.

**Figure 7:**
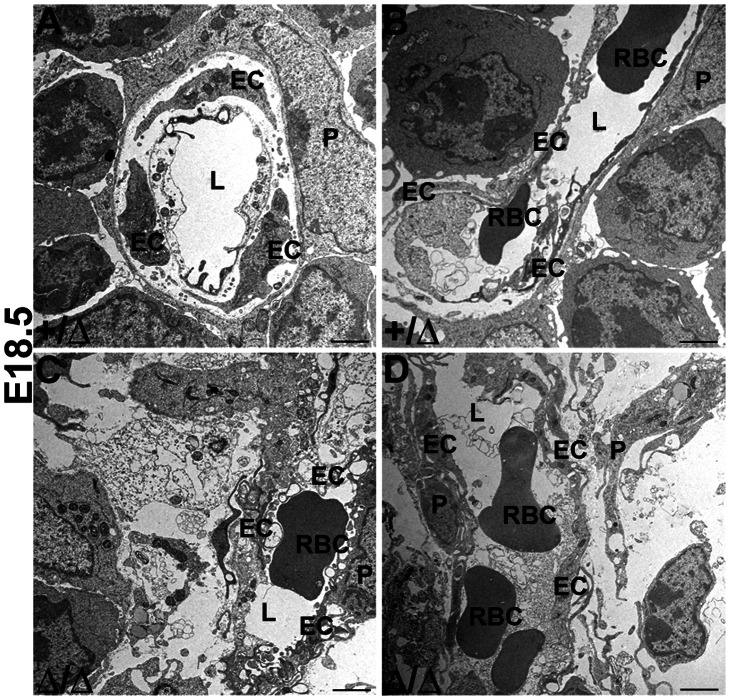
Electron Microscopic Analysis of E18.5 thymus vascular defects. Electron microscopy analysis of (**A–B**) *Foxn1^+^* ^/*Δ*^ thymus show compact arrangement of cells including endothelial cells and pericytes, while the (**C–D**) *Foxn1^Δ^* ^/*Δ*^ thymus display loose arrangement of cells, vacuolated endothelium, and indistinct vessel walls. Endothelial Cell (**EC**), Pericyte (**P**), Lumen of Blood Vessel (**L**), and Red Blood Cell (**RBC**) n = 3.

To investigate the origin of the ‘leaky’ vasculature phenotype identified by FITC-dextran injection (Figure 5B, D, F, H), we also assessed whether mature vessels with perivascular cell coverage developed in the *Foxn1* ^Δ*/*Δ^ thymus, based on a morphological analysis of endothelial cell-pericyte interactions. We observed pericytes associated with most vascular structures in the thymus (Figure 7A–D). *Foxn1^+/^* ^Δ^ thymi showed normal endothelial morphology and a compact arrangement of endothelium and pericytes (Figure 7A–B). In contrast, *Foxn1* ^Δ*/*Δ^ thymi showed vacuolated endothelium, edema, indistinct vessel walls, and an overall loose arrangement of cells due to separation and cellular swelling (Figure 7C–D). These results indicate that while endothelial cells in the *Foxn1* ^Δ*/*Δ^ thymus are competent to recruit perivascular cells, endothelium-pericyte interactions were altered and vascular patterning was defective in *Foxn1* ^Δ*/*Δ^ thymi.

### VEGF-A and PDGF-B Levels are Reduced in Foxn1^Δ/Δ^ Mice

**Figure 8:**
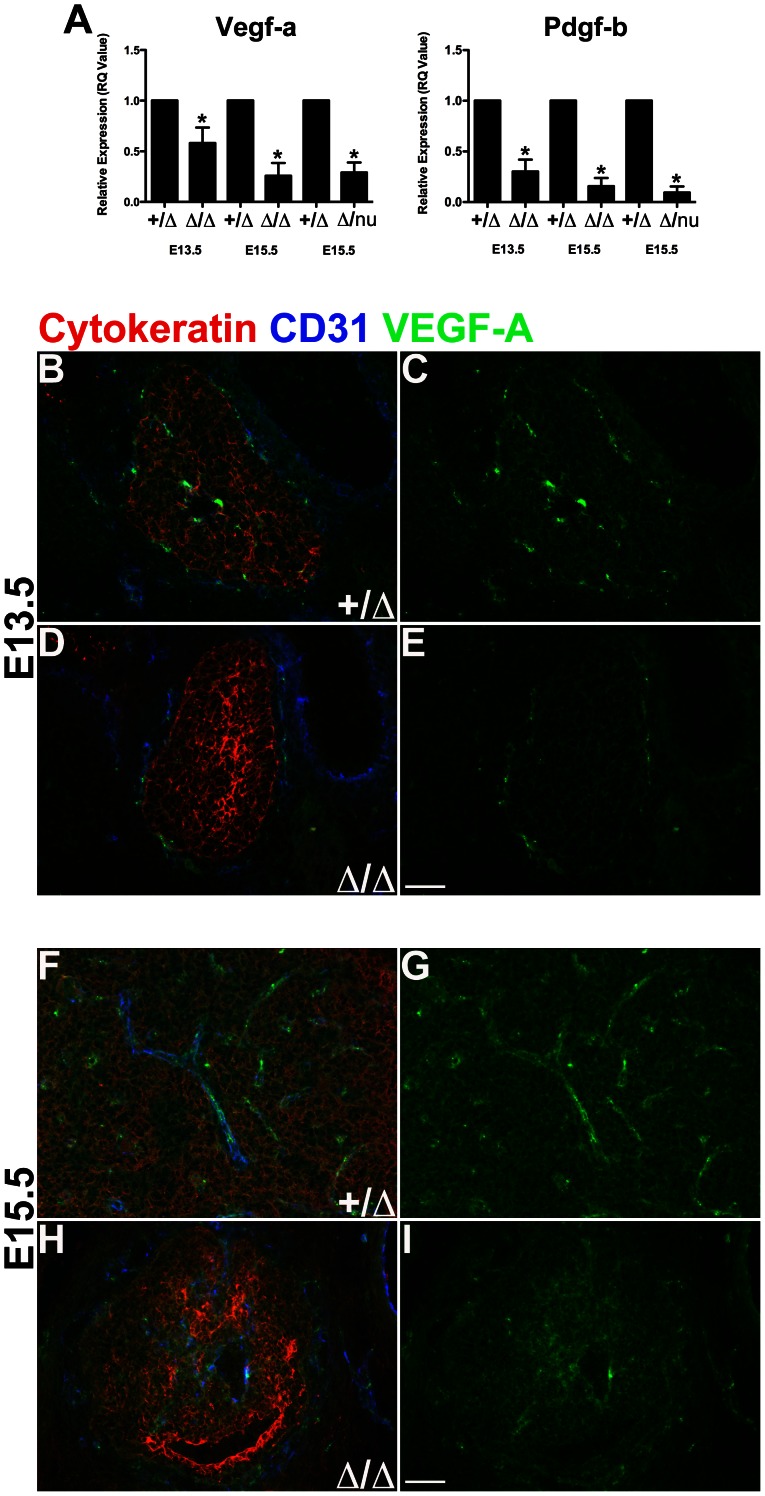
VEGF-A and PDGF-B expression reduced in *Foxn1^Δ/Δ^* thymus. (**A**) VEGF-A expression was significantly reduced in E13.5 *Foxn1^Δ^* ^/*Δ*^ **(**n = 4), E15.5 *Foxn1^Δ^* ^/*Δ*^ (n = 3), and E15.5 *Foxn1^Δ^* ^/nu^ (n = 6), compared to *Foxn1^+^* ^/*Δ*^ control thymi. PDGF-B expression was also reduced in E13.5 *Foxn1^Δ^* ^/*Δ*^ (n = 4), E15.5 *Foxn1^Δ^* ^/*Δ*^ (n = 3), and E15.5 *Foxn1^Δ^* ^/nu^ (n = 6), compared to *Foxn1^+^* ^/*Δ*^ control thymi. Experiments represent relative RNA expression of pooled thymi. Controls were set to 1. Asterisks denote statistical significance (P<0.05). (**B–E**) Immunofluorescence analysis of VEGF-A expression performed on frozen transverse sections of embryonic thymus for CD31^+^ (**blue**), VEGF-A (**green**) and Cytokeratin (**red**). VEGF-A expression was detected in thymic endothelium, perivascular cells, and TECs in *Foxn1^+^* ^/*Δ*^ and *Foxn1^Δ^* ^/*Δ*^ mice (**B–E**). VEGF-A expression was reduced in (**D–E**) E13.5 *Foxn1^Δ^* ^/*Δ*^ thymus compared to (**B–C**) E13.5 *Foxn1^+^* ^/*Δ*^ controls. Scale bar, 100 µm; n = 3 or more.

We next tested whether defects in Foxn1-dependent TEC differentiation affect VEGF-A and PDGF-B expression in the thymus. Vascular endothelial growth factor (VEGF) is a potent inducer of vascular development during embryogenesis and in adults [37], [38], [39], [40]. In the thymus, VEGF-A expression has been reported in TECs, NCCs, endothelial cells, and a subset of immature thymocytes [19], [20]. We measured mRNA levels in thymic stromal cells of *Foxn1* ^Δ*/*Δ^, *Foxn1* ^Δ*/nu*^ and control littermates. VEGF-A (Figure 8A) expression was significantly reduced (p<0.05) at both E13.5 and E15.5 in *Foxn1* ^Δ***/***Δ^ and at E15.5 *Foxn1* ^Δ*/nu*^ mice compared to control littermates. By immunofluorescence, VEGF-A protein was predominantly associated with vasculature, and TECs (Figure 8B–I). In E13.5 *Foxn1* ^Δ*/*Δ^ thymi, which do not have vasculature inside the thymus, VEGF-A protein staining was highest in the capsule vasculature, but virtually undetectable in TECs (Figure 8D–E). By E15.5, VEGF-A clearly delineates the vasculature in controls (Figure 8F–G) but not in *Foxn1* ^Δ*/*Δ^ thymi (Figure 8H–I), indicating that the decreased VEGF-A expression may be at least in part from the vascular-associated mesenchyme and/or endothelium.

PDGF-B expression was also reduced at E13.5 and E15.5 in *Foxn1* ^Δ*/*Δ^ and at E15.5 in *Foxn1* ^Δ*/nu*^ thymic stromal cells by qRT-PCR (Figure 8A). PDGF-B is expressed by endothelial cells and functions in the recruitment of mesenchyme to developing vasculatures, thus providing structural support and necessary growth/survival factors required for vessel homeostasis [41], [42], [43]. Thus, this down regulation could be an indirect consequence of delayed immigration of vasculature and reduced endothelial cell numbers in the mutant thymus.

### Collagen IV is Present throughout the Foxn1^Δ/Δ^ Thymus

Collagen IV is a major component of the mammalian basement membrane and extracellular matrix (ECM) and is critical for cell migration, adhesion, proliferation, and differentiation [44]. In the thymus, collagen IV is used as a marker of perivascular spaces that are the location of lymphocyte immigration and egress [45], [46]. It is deposited by growing blood vessels, and the presence of ‘empty’ collagen IV^+^ sleeves has been demonstrated as an indication of abnormal vascular development in tumors. We examined ontogeny of collagen IV deposits in wild-type thymus to investigate whether the cell autonomous defects in *Foxn1* ^Δ*/*Δ^ TECs resulted in subsequent defects in collagen IV deposition and perivascular space formation [47]. In E12.5 *Foxn1^+/^* ^Δ^ thymi, collagen IV was localized to the capsular region (Figure S3). Following initial thymic vascularization, collagen deposits were primarily associated with CD31^+^ cells, although there were some examples of CD31^−^ collagen IV^+^ beds (Figure S3). In newborn *Foxn1^+/^* ^Δ^ thymi, nearly all collagen IV deposits were closely associated CD31^+^ cells in the cortex and medulla (Figure S3). There were no discernable differences in collagen IV deposition in E12.5–E15.5 *Foxn1* ^Δ*/*Δ^ compared to control thymi (Figure S3A–F). In contrast, by the newborn stage, collagen IV was very broadly localized throughout the *Foxn1* ^Δ*/*Δ^ thymus (Figure S3H), with CD31^+^ collagen IV^+^ structures throughout the mutant thymus compared with littermate controls (Figure S3G), further indicating that vascular development in the *Foxn1* ^Δ*/*Δ^ thymus is abnormal.

## Discussion

In this study, we have defined the early stages of thymic vascularization during organogenesis in the mouse, and established that Foxn1-dependent TEC differentiation is required for this process. Specifically, our data demonstrate that Foxn1 function in TECs is required to regulate the initial formation of the thymic vasculature, and that this function is sensitive to Foxn1 function and dosage. Effects on vascular development are non-cell-autonomous, and may be due to either direct production of angiogenic factors by TECs, or to more indirect effects of reduced thymus size. Vasculature initially infiltrates the thymus at E13.5, followed closely by PDGFR-β^+^ mesenchyme; these immigration events were specifically delayed by one day in *Foxn1* ^Δ*/*Δ^ mice. Further, we demonstrated that once formed, the organization and structure of the thymic vasculature in Foxn1^Δ/Δ^ mice is abnormal, with changes in both vessel size and structural integrity that indicate defective maturation of and interactions between endothelial and mesenchymal cells. In contrast, the timing of initial LPC immigration into the *Foxn1* ^Δ*/*Δ^ fetal thymus was normal, although as reported previously, the number of thymocytes was significantly reduced due to delayed and reduced proliferation and differentiation [27]. We have also shown that the peripheral circulation connects to the thymic rudiment as early as E14.5, and that the timing of this process is normal in *Foxn1* ^Δ*/*Δ^ mutants. Since not all events during early thymus organogenesis are delayed, these defects may reflect specific functions for TECs in regulating vascularization, rather than a general delay in thymus development. The overall reduction in thymus size in *Foxn1* ^Δ*/*Δ^ mice may also contribute to the observed defects in thymus vascularization. TEC specific deletions of pro-angiogenic molecules will be needed to establish specific roles for TECs in driving the formation of the thymus vasculature. The non-cell-autonomous nature of the vasculature-associated defects in the *Foxn1* ^Δ*/*Δ^ mutants provides evidence for a TEC-endothelium-mesenchyme interaction that plays a key role in thymus organogenesis and the organization of the thymic architecture.

Taken together, our current results and those of previous studies suggest the following model for initial thymic vascularization. Neural crest cells are first recruited to the periphery of the thymus at E10.5–E11.5 [16], [48]. Subsequently, vasculature is attracted to the pericapsular region of the thymus at E12.5. The data suggest that between E12.5–E13.5 (Figure 2D, I), sprouting endothelium invades the mesenchymal capsule and infiltrate the thymus ahead of NCCs (although we cannot exclude the possibility of individual endothelial cells immigrating into the thymus). One day later at E14.5, capsular NCCs form invaginations into the TEC network and migrate along the path of nascent blood vessels to form perivascular support cells (Figure 2K–N) [17]. At the same time, the developing intrathymic vasculature becomes physically connected to the peripheral fetal vasculature, further supporting the conclusion that E14.5 is the earliest stage at which the fetal thymic vasculature becomes functional. Thus, the thymic vasculature likely forms by the coordination of intrathymic vasculogenesis (controlled in part by signals from TECs directly or indirectly downstream of Foxn1) and extrathymic angiogenesis (the timing of which is independently regulated).

This cellular order of vascular development, the *Foxn1* ^Δ*/*Δ^ phenotype, and the expression and function of PDGF and VEGF further suggest a molecular model for the mechanisms controlling this process. Both NCCs [49] and TECs [19], [20] have been reported to express pro-angiogenic factors that may facilitate recruitment of the vasculature to the capsular region of the thymus, and subsequently into the thymic anlage. As initial vasculature recruitment to the thymus capsule is not affected in the *Foxn1* ^Δ*/*Δ^ mutants, this process may be regulated either by NCC-derived signals, or by Foxn1-independent TEC-based signals, while immigration into the rudiment itself may require TEC-derived signals downstream of Foxn1. Once inside the rudiment, branching vasculature structures and possibly angioblasts form the intrathymic vasculature via vasculogenesis. The structure of this network is patterned by the level of intrathymic proangiogenic molecules [19], [20]. This intrathymic network is connected to the peripheral vasculature at E14.5 via invasion of smaller vessels from outside the thymus; the timing of this process is independent of Foxn1-dependent TEC-derived signals, and may be regulated by mesenchymal capsule-derived angiogenic factors. Earlier injections were ambiguous due to high background; this result may have a biological rather than technical origin, as the NCC-derived mesenchyme does not become fully associated with the intrathymic vasculature until E14.5. Thus, earlier time points would not likely have a functional vasculature even in wild-type thymi.

Although reports suggest VEGF-A mRNA is expressed in TECs [19], [20], we were unable to detect high levels of VEGF-A protein in TECs (Figure 8); it is possible that VEGF is being further regulated at the translational or protein level. Instead, VEGF protein was concentrated in the endothelial cells in the thymic vasculature; this vascular-associated VEGF was clearly reduced in *Foxn1* ^Δ*/*Δ^ mice. Interestingly, E15.5 *Foxn1* ^Δ*/nu*^ mice had a similar level of VEGF-A as *Foxn1* ^Δ*/*Δ^ mice, but show more severe vascular defects. The loss of other TEC-derived pro-angiogenic factors in TECs might account for this difference. Alternatively, the more severe phenotype may reflect other changes in TEC differentiation that could affect the accessibility of the thymic epithelial rudiment to invasion by the vasculature.

Our data suggest that the loss of both VEGF-A and PDGF-B expression are due to a non-cell autonomous effect, by disruption of TEC-endothelial cell interactions, which in turn disrupts endothelial-NCC interactions. Alternatively, TECs could interact directly with both endothelial cells and NCCs. Our results also suggest that NCC-derived mesenchyme “follow” the endothelium into the thymus, supporting a previous report indicating that nearly all NCC-derived mesenchyme within the adult thymus is associated with blood vessels [17]. In the *Foxn1* ^Δ*/*Δ^ mutants, both the delay in mesenchymal cell immigration and the reduced intrathymic PDGF-B expression are likely secondary to the delayed and reduced number of intrathymic endothelium. Endothelial cell-mural cell contact is predominantly mediated by PDGFR-β/PDGF-B ligand/receptor signaling [41], [42], [50], [51]. During normal fetal thymus development and in adult thymi, greater than 90% of NCCs express PDGFR-β [17], which suggests that thymic endothelial cell PDGF-B expression facilitates NCC recruitment into the organ. These results are consistent with the established model for endothelium-mesenchyme paracrine signaling [52]. PDGFR-β levels in *Foxn1* ^Δ*/*Δ^ mutants were also reduced (Figure 1), either in response to reduced ligand availability, or as a consequence of defective TEC-NCC interactions. TECs could also directly affect NCC behavior either expressing PDGF-B (although previous reports do not support this possibility) [41], [42], [43], PDGF-A, or providing other factors that promote mesenchymal migration and/or differentiation. While no defects were reported in thymic vasculature in *PDGFRβ^−/−^* mutants [17], the integrity of the vasculature and initial timing of thymus vascularization were not examined. Thus, while reduced PDGF-B levels in the thymus may contribute to delayed pericyte recruitment to blood vessels, a redundant signaling pathway(s) may compensate in the absence of PDGF-B.

The TEC differentiation and mTEC organization phenotypes in *Foxn1* ^Δ*/*Δ^ mice were observed as early as E13.5 [27], coincident with the initial vasculature immigration delay reported here. A previous study suggested that the thymic vasculature may play a role in organizing mTECs independent of thymocyte-TEC crosstalk, based on the spatial arrangement between the thymic vasculature and mTECs in wild-type and *Rag^−/−^* mutant mice [10]. The combination of vascular and stromal organization defects during early thymus organogenesis in the *Foxn1* ^Δ*/*Δ^ mutants raises the possibility that some of the thymic stromal organization defects observed in *Foxn1* ^Δ*/*Δ^ thymi may, in part, be secondary to the early defects in initial thymic vascular development. Furthermore, the localization of collagen IV in the thymus normally delineates the perivascular space (PVS), where mature T-cells accumulate prior to immigration into the thymus and egress to the periphery [53]. As an increased number of immature T-cells are present in the periphery of adult *Foxn1* ^Δ*/*Δ^ mice [28], the broad distribution of collagen IV in these mutants could allow thymocytes to exit the thymus prematurely through blood vessels positioned throughout the organ.

The paradigm of TEC-thymocyte crosstalk informs much of our understanding of the mechanisms underlying the organization and function of the thymus. The identification of further interactions between TECs, endothelial cells, and mesenchymal cells expands the network of intercellular interactions required for the proper formation of the complex thymic architecture. Our current and previous data suggest that Foxn1 plays a central role in TECs to orchestrate these diverse crosstalk pathways during fetal thymus organogenesis. This novel TEC-endothelium-NCC interaction may also be required for the cortico-medullary organization, and for formation of blood vessels at spatially defined regions of the thymus that facilitates the immigration of LPCs into the thymus and egress of LPCs to the periphery.

## Supporting Information

Figure S1 **EpCAM and CD45 expression in depleted stroma from** ***Foxn1^Δ/Δ^*** **thymus.** (**A**) EpCAM expression is normal in pooled E13.5 *Foxn1^Δ^* ^/*Δ*^ and control thymi before and after CD45^+^ cell depletion. (**B**) CD45 expression before and after CD45^+^ cell depletion in pooled E13.5 *Foxn1^Δ^* ^/*Δ*^ and control thymi.
(TIF)

Figure S2 **Initial thymic vascularization is similar in E13.5** ***Foxn1^+/+^*** **and** ***Foxn1^+/Δ^*** **mice.** Immunofluorescence analysis of CD144 (VE-Cadherin) and Keratin 5 (K5) in the wild-type thymus from frozen sections of whole embryos (E12.5, E13.5) and dissected thymi (E15.5, E17.5). Timing of vascularization of overall vessel patterning is similar to that seen in *Foxn1^+^* ^/*Δ*^ mice, as shown in Figure 2.
(TIF)

Figure S3 **Collagen IV is broadly expressed throughout NB** ***Foxn1^Δ/Δ^*** **thymus.** Immunofluorescence analysis of embryonic (**A–F**) and newborn transverse sections (**G–H**) of thymus for CD31^+^ (**blue**) and Collagen IV (**green**). Collagen IV deposits adjacent to CD31^+^ cells in E12.5 (**A**) *Foxn1^+^* ^/*Δ*^ and (**B**) *Foxn1^Δ^* ^/*Δ*^, E13.5 (**C**) *Foxn1^+^* ^/*Δ*^ and (**D**) *Foxn1^Δ^* ^/*Δ*^, E15.5 (**E**) *Foxn1^+^* ^/*Δ*^ and (**F**) *Foxn1^Δ^* ^/*Δ*^, Collagen IV expression in newborn (**G**) *Foxn1^+^* ^/*Δ*^ and (**H**) *Foxn1^Δ^* ^/*Δ*^ thymus. Cortex (**c**) and medulla (**m**) Scale bar, 100 µm; n ≥ 3.
(TIF)
