# Supplementary figures and images for: Cell-Autonomous Defects in Thymic Epithelial Cells Disrupt Endothelial-Perivascular Cell Interactions in the Mouse Thymus
Source: PLoS One. 2013 Jun 4;8(6):e65196. doi: 10.1371/journal.pone.0065196 (PMC3672159; doi:10.1371/journal.pone.0065196)

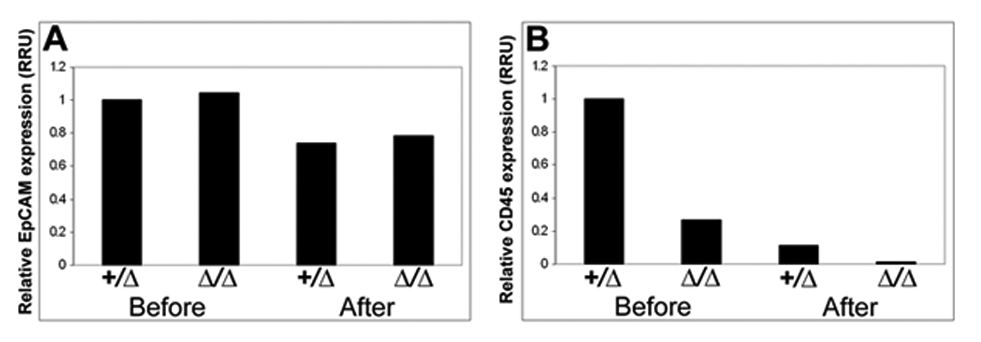

Supplement: Figure S1 — EpCAM and CD45 expression in depleted stroma from Foxn1Δ/Δ thymus. (A) EpCAM expression is normal in pooled E13.5 Foxn1Δ /Δ and control thymi before and after CD45+ cell depletion. (B) CD45 expression before and after CD45+ cell depletion in pooled E13.5 Foxn1Δ /Δ and control thymi. (TIF) [file pone.0065196.s001.tif]

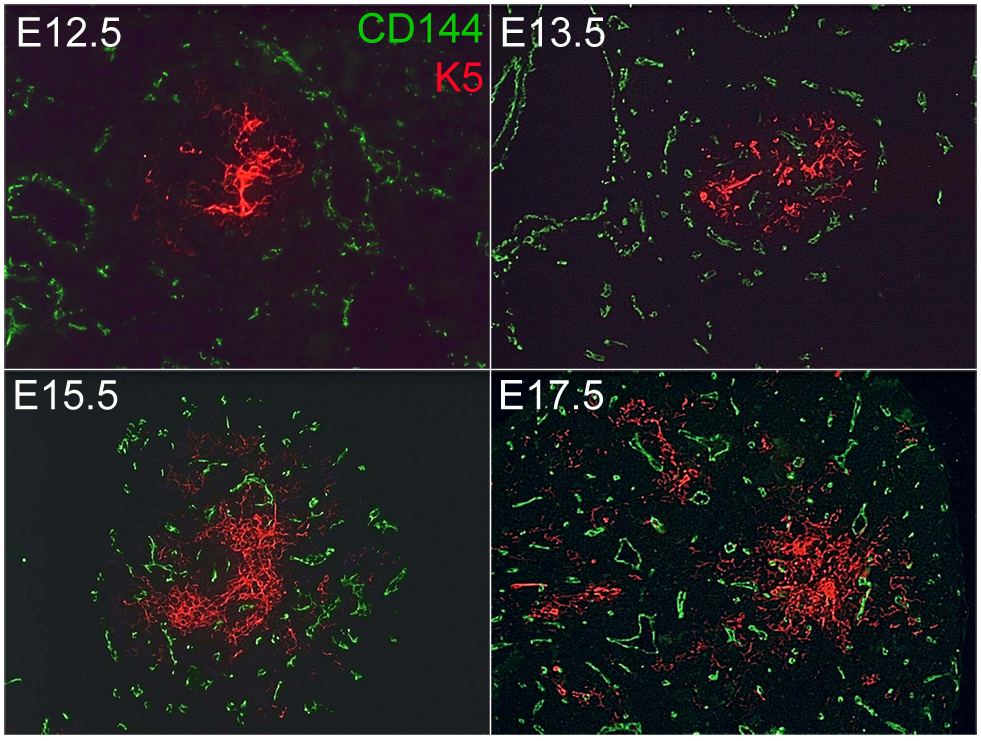

Supplement: Figure S2 — Initial thymic vascularization is similar in E13.5 Foxn1+/+ and Foxn1+/Δ mice. Immunofluorescence analysis of CD144 (VE-Cadherin) and Keratin 5 (K5) in the wild-type thymus from frozen sections of whole embryos (E12.5, E13.5) and dissected thymi (E15.5, E17.5). Timing of vascularization of overall vessel patterning is similar to that seen in Foxn1+ /Δ mice, as shown in Figure 2. (TIF) [file pone.0065196.s002.tif]

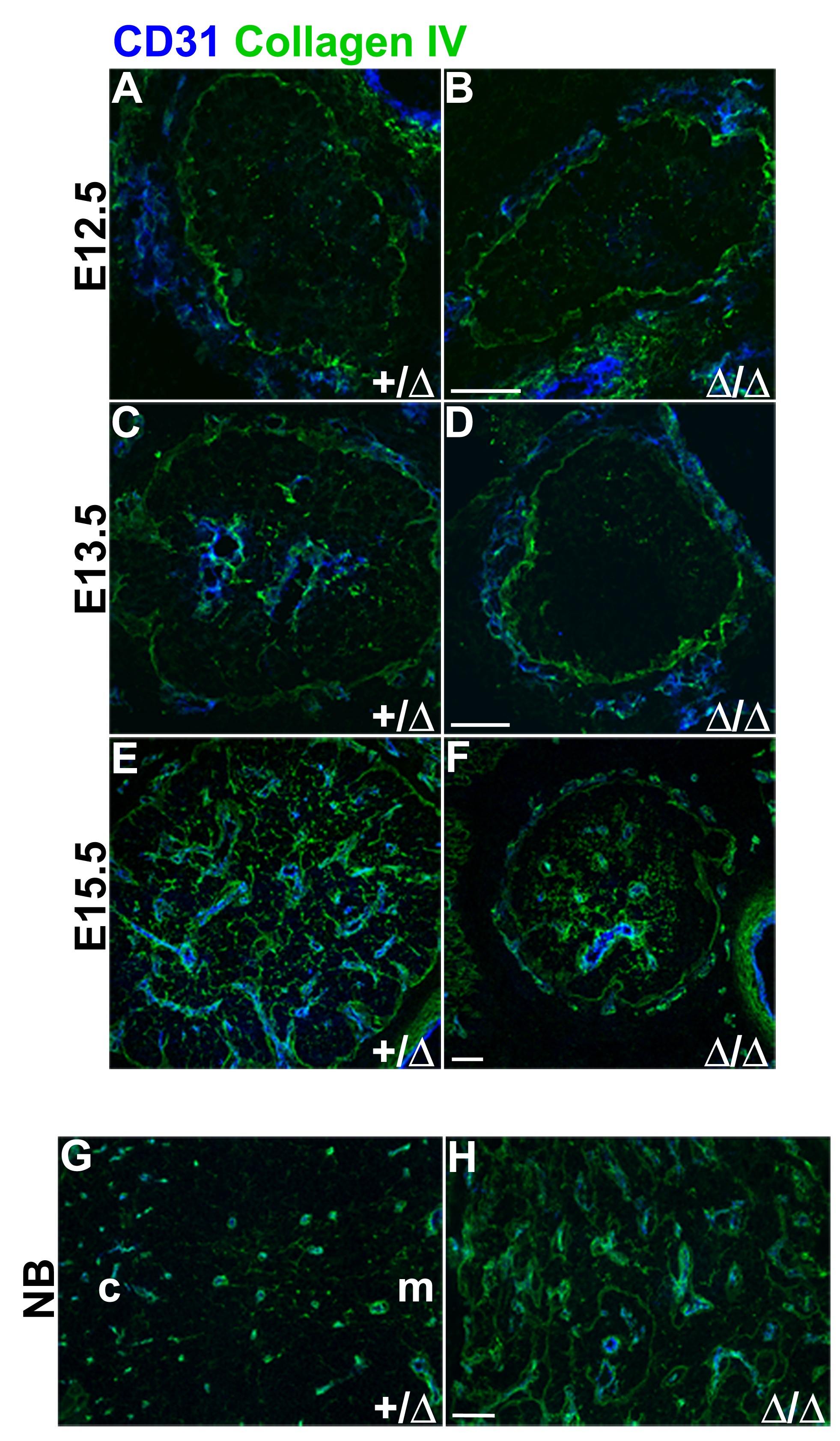

Supplement: Figure S3 — Collagen IV is broadly expressed throughout NB Foxn1Δ/Δ thymus. Immunofluorescence analysis of embryonic (A–F) and newborn transverse sections (G–H) of thymus for CD31+ (blue) and Collagen IV (green). Collagen IV deposits adjacent to CD31+ cells in E12.5 (A) Foxn1+ /Δ and (B) Foxn1Δ /Δ, E13.5 (C) Foxn1+ /Δ and (D) Foxn1Δ /Δ, E15.5 (E) Foxn1+ /Δ and (F) Foxn1Δ /Δ, Collagen IV expression in newborn (G) Foxn1+ /Δ and (H) Foxn1Δ /Δ thymus. Cortex (c) and medulla (m) Scale bar, 100 µm; n ≥ 3. (TIF) [file pone.0065196.s003.tif]
